# Advances and roadblocks in the treatment of malaria

**DOI:** 10.1111/bcp.14474

**Published:** 2020-08-01

**Authors:** Borimas Hanboonkunupakarn, Nicholas J. White

**Affiliations:** ^1^ Faculty of Tropical Medicine Mahidol University Bangkok Thailand; ^2^ Centre for Tropical Medicine and Global Health, Nuffield Department of Medicine Oxford University UK

**Keywords:** antimalarial drugs, artemisinin, malaria, resistance

## Abstract

The deployment of artesunate for severe malaria and the artemisinin combination therapies (ACTs) for uncomplicated malaria has been a major advance in antimalarial therapeutics. These drugs have reduced treated mortality, accelerated recovery and reduced treatment failure rates and transmission from the treated infection. Artemisinin derivatives remain highly effective against falciparum malaria in most malaria endemic areas, but significant resistance has emerged in the Greater Mekong subregion of Southeast Asia. Resistance to artemisinins was followed by resistance to the ACT partner drugs, and fit multidrug resistant parasite lineages have now spread widely across the region. ACTs remain highly effective against *P. vivax* and the other malaria species. Recent studies have shown that radical curative regimens of primaquine (to prevent relapse) can be shortened to 7 days, and that the newly introduced single dose tafenoquine is an alternative, although the currently recommended dose is insufficient in Southeast Asia and Oceania. Targeted malaria elimination using focal mass treatments with dihydroartemisinin‐piperaquine have proved safe and effective malaria elimination accelerators, but progress overall towards malaria elimination is slow. Indeed since 2015 overall malaria case numbers globally have risen. As new drugs will not become widely available in the near future, active measures to preserve the current antimalarials should be given the highest priority.

## 1 INTRODUCTION

The treatment of malaria has improved substantially in the past 15 years, and morbidity and mortality have declined as a result, but significant challenges lie ahead.^1^ The major advance in antimalarial therapeutics has been the deployment of drugs derived from artemisinin (qinghaosu).^2^ This unusual compound (a sesquiterpene lactone peroxide) is derived from the leaves of the plant *Artemesia annua.* The derivatives of artemisinin, dihydroartemisinin (DHA), artesunate and artemether, now form the cornerstone of current antimalarial treatment. They are the most rapidly acting of the available antimalarial drugs and they are very well tolerated, but resistance has now emerged in Southeast Asia, and it has spread, and there are worrying early reports of foci in other regions. The artemisinins are partnered in fixed‐dose combinations (artemisinin combination therapies, ACTs) with more slowly eliminated antimalarials for the treatment of uncomplicated malaria. New antimalarial drugs are on the horizon, but they are unlikely to become generally available within the next few years, so treatments now and in the immediate future must rely upon the artemisinin derivatives. This review presents some of the recent advances in antimalarial therapeutics and some of the obstacles to progress in controlling and eliminating malaria.

### 1.1 Advance 1: Improvements in the treatment of severe malaria

**FIGURE 1:**
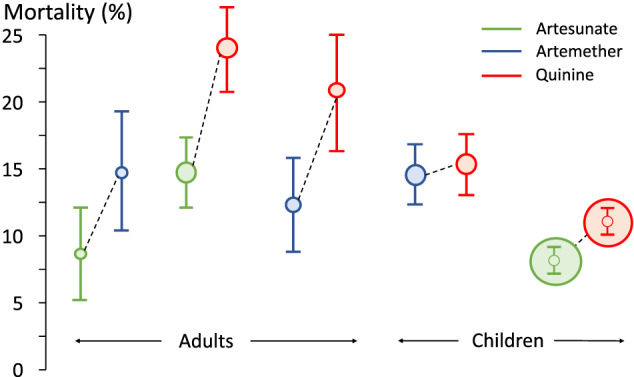
Mortality by treatment arms in randomized comparative controlled trials in strictly defined severe falciparum malaria (which together enrolled 2874 adults and 7424 children). The size of the circle is approximately proportional to the size of the trial and the error bars are 95% confidence intervals. The adults were enrolled mainly in Southeast Asia and the children mainly in Africa^3^, ^4^, ^5^, ^6^

In the two largest randomized controlled trials conducted in patients hospitalized with severe falciparum malaria artesunate was shown to reduce mortality substantially. Compared to parenteral quinine, the previous first‐line treatment, the mortality reduction (95% confidence interval) was 34.7% (18·5‐47·6%) in Southeast Asian adults and children and 22.5% (8·1‐36·9%) in African children^3^, ^4^ (Figure 1). In African children it can be difficult to distinguish severe malaria from bacterial sepsis with incidental parasitaemia. In those children with a high likelihood of having severe malaria, based on parasite biomass estimation, the reduction in mortality was the same (ie, one‐third) as observed in the Asian series.^7^ Artesunate was not more expensive than parenteral quinine, it was better tolerated (less hypoglycaemia), and it was easier to administer (intravenous injection rather than controlled rate infusion, and no pain or local toxicity following intramuscular injection). Importantly there were also fewer neurological sequelae in the survivors, so lives were not saved by artesunate at the expense of neurological deficits. Artemether is nearly as active as artesunate in vitro against *Plasmodium falciparum* but, being an oil‐based intramuscular injection, is slowly and erratically absorbed from the intramuscular injection site in vivo (particularly in shocked patients).^8^ In contrast the water‐soluble artesunate is rapidly and reliably absorbed after intramuscular injection. This delay in reaching parasiticidal blood concentrations probably explains why the severe malaria mortality following artemether in randomized trials was higher than that following artesunate treatment^5^, ^6^ (Figure 1). Community based pre‐referral rectal artesunate was also shown to reduce malaria attributable mortality by 25% in children who were unable to take oral antimalarial medications.^9^ Since these trials reported over 10 years ago parenteral artesunate has become the generally recommended first‐line treatment for severe malaria^1^ and usage has increased substantially, although unfortunately quinine is still the only available parenteral antimalarial in some malaria endemic areas.

Following drug administration the artemisinin derivatives are rapidly and reliably converted back to DHA in vivo, which is then eliminated very rapidly (*t*_1/2_ ≈ 1 hour), mainly by glucuronidation.^10^ Despite this the drugs are highly efficacious when given once daily.^11^ The main pathological process in severe falciparum malaria is the sequestration of erythrocytes containing mature forms of the parasite in the vascular beds of vital organs.^7^, ^12^ This reduces microcirculatory blood flow and probably markedly disturbs endothelial function. The key pharmacodynamic advantage of the artemisinin derivatives, which mediates their life‐saving advantage over quinine, is the killing of the younger circulating stages of *P. falciparum* before they sequester.^12^ Unfortunately, this property is substantially reduced in artemisinin resistance (see below Obstacle 2: Artemisnin resistance).

Apart from the prompt initiation of renal replacement therapies (preferably haemofiltration) in acute kidney injury,^12^, ^13^ no adjuvant therapies in severe malaria have proved to be beneficial, and many (including aspirin, corticosteroids, heparin, mannitol, high dose phenobarbitone, anti‐TNF antibody and rapid fluid loading) were found to be harmful.^12^, ^14^

### 1.2 Advance 2: Better treatments for uncomplicated falciparum malaria

**FIGURE 2:**
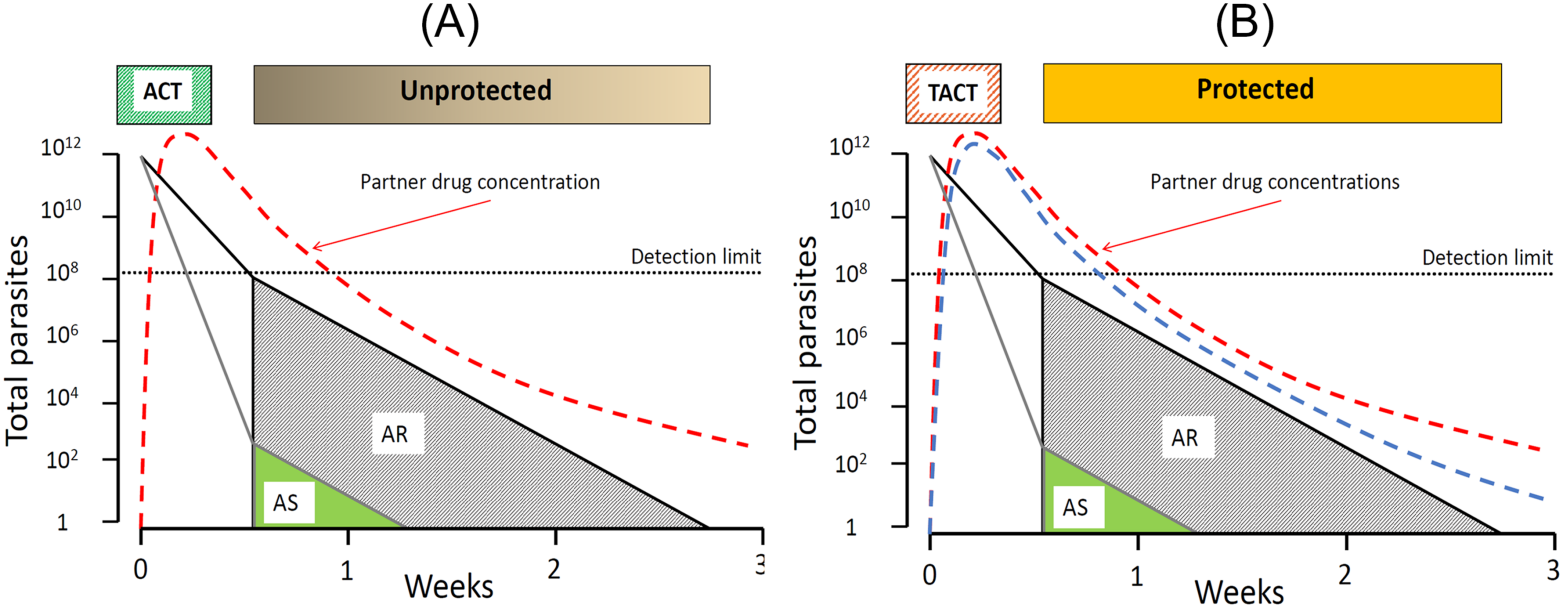
In artemisinin‐sensitive malaria infections (AS) the 3‐day artemisinin regimen in ACTs (A), results in rapid parasite killing and consequent decline in parasitaemia. The logarithmic scale vertical axis shows the total number of malaria parasites in the body of an adult with approximately 2% parasitaemia. The ACT partner drug has only approximately 1000 parasites to remove in this example (green triangle). In contrast, in an artemisinin‐resistant infection (AR) there is substantially less parasite killing initially and so the ACT partner drug now has approximately 100 million parasites to remove (alone) with a substantially greater risk of treatment failure (recrudescence) and thus selective pressure to the emergence of partner drug resistance. In B, with TACTs there are now two slowly eliminated drugs providing a potentially greater antimalarial effect in resistant infections and ensuring mutual protection against the emergence of resistance. The detection limit (black dotted line) is the limit for microscopy to identify a malaria infection

The main advance in the treatment of uncomplicated falciparum malaria has been the replacement of the failing monotherapies chloroquine and sulfadoxine‐pyrimethamine by artemisinin combination therapies (ACTs). This process began in earnest about 15 years ago.^1^ These 3‐day ACT regimens combine an artemisinin derivative with a more slowly eliminated partner drug (Figure 2A). Four ACTs were recommended originally; artesunate combined with either sulfadoxine‐pyrimethamine (SP), amodiaquine or mefloquine, and artemether combined with lumefantrine. More recently dihydroartemisinin‐piperaquine and artesunate‐pyronaridine have been added.^1^, ^15^ All except artesunate‐SP are available in combined formulations, and all but artemether‐lumefantrine are taken once daily. These drugs are rapidly effective and generally well tolerated.^1^, ^10^, ^16^ Early concerns over potential neurotoxicity and teratogenicity have receded with increasing evidence of safety.^12^ Worries over piperaquine cardiotoxicity (QT prolongation, risk of Torsade de Pointes) have also declined, with a large meta‐analysis showing no increase in the rate of sudden death.^17^ ACTs are now recommended as first‐line treatments for all patients with falciparum malaria, including in pregnancy.^1^ They are an alternative to chloroquine for infections caused by the other malaria species, allowing a single treatment to be deployed for all malaria infections. Costs have been reduced and generics developed. Hundreds of millions of treatments are dispensed annually.

The main current concerns are ensuring access to diagnosis and effective treatment, and containing emerging resistance in *P. falciparum* to the artemisinin derivatives. Artemisinin resistance (see below Obstacle 2: Artemisinin resistance) manifests as slowing of parasite clearance because of reduced ring stage (the younger asexual forms) parasite susceptibility.^18^ The discovery of a molecular marker, mutations in the propeller region of the *P. falciparum* kelch gene on chromosome 13, has greatly facilitated characterization and epidemiological assessments.^19^, ^20^ Reduced parasite killing in artemisinin‐resistant malaria infections places greater selective “pressure” on the ACT partner drug. This is because the number of parasites which remain in the circulation after the artemisinin component in an ACT has been eliminated is many orders of magnitude greater, and the probability of selecting resistant mutants is correspondingly higher (Figure 2A). Indeed, ACT partner drug resistance has followed artemisinin resistance in the Greater Mekong subregion (GMS) of Southeast Asia.^21^, ^22^, ^23^, ^24^ Fortunately artemisinin‐resistant *P. falciparum* are still largely confined to this one region,^25^ although there are increasing reports that clusters of kelch mutant parasites have been identified elsewhere.^26^, ^27^ One potential therapeutic solution, which could be deployed now, is to combine an artemisinin derivative with two slowly eliminated antimalarials (triple artemisinin combination treatments, TACTs).^23^ This solves the pharmacokinetic mismatch whereby the rapidly eliminated artemisinin component leaves the slowly eliminated partner drug “unprotected” for days or weeks after the second post‐treatment asexual parasite cycle (ie, >3 days after starting the ACT). With TACTs there are now two slowly eliminated partner drugs providing mutual protection against the selection of resistance (Figure 2B). The two TACTs under current development, artemether‐lumefantrine‐amodiaquine and dihydroartemisinin‐piperaquine‐mefloquine, exploit fortuitous reciprocal susceptibilities whereby resistance to one of the slowly eliminated components is associated with increased susceptibility to the other. In large‐scale trials TACTs have proved well tolerated, safe and highly effective.^24^

### 1.3 Advance 3: Chemoprevention in malaria endemic areas

For many years pregnant women living in a malaria endemic region were advised to take chloroquine chemoprophylaxis to reduce the adverse effects of falciparum malaria on the developing foetus (mainly low birthweight). Then, as chloroquine resistance worsened, chemoprophylaxis in Africa was replaced by intermittent presumptive treatment with sulphadoxine‐pyrimethamine (IPT‐SP).^1^ This imperfect chemoprophylaxis involves giving full treatment doses at intervals of 1 month or more. Although preventive efficacy is much greater than treatment efficacy against resistant *P. falciparum* , IPT‐SP is threatened by worsening resistance, both to the antifol and the sulphonamide components.^28^ There is increasing evidence that dihydroartemisinin‐piperaquine (DP) provides excellent antimalarial chemoprevention for approximately 1 month, is well tolerated and appears to be safe in pregnancy.^29^, ^30^ To provide continuous suppressive prophylaxis DP needs to be given at least monthly and preferably weekly.^31^ The IPT‐SP concept has also been advocated in infants, where treatment doses of SP are to be given together with the routine expanded programme on immunization (EPI) vaccines at the ages of 2, 3 and 9 months. This is not widely practiced as the benefits are relatively small and SP resistance is widespread. A more effective strategy, which is now implemented widely across the Sahel region of Africa (where intense malaria transmission is largely confined to the 3‐4‐month rainy season), is seasonal malaria chemoprevention (SMC). This is monthly administration of treatment doses of amodiaquine together with SP to all children aged between 6 and 59 months.^1^, ^32^ SMC prevents symptomatic reinfections and substantially reduces the malaria burden. Mass drug administrations with azithromycin have been associated with reduced all‐cause mortality in African children, particularly in Niger.^33^ However, adding azithromycin to SMC with amodiaquine and SP in West Africa was shown to provide no additional benefit.^34^ Resistance to both components of SMC is widespread in East Africa, but whether resistance is impacting on the chemoprophylactic activity of SMC is uncertain currently. More information is needed on this critical point to guide policy.

### 1.4 Advance 4: Mass treatment as a malaria elimination accelerator

Where malaria transmission is low, the prospects for elimination increase. In the GMS, an area of low seasonal malaria transmission which harbours the most drug‐resistant *P. falciparum* in the world, there is a general consensus that the only way to counter multidrug resistance effectively is to eliminate all falciparum malaria. The urgency to counter the threat of artemisinin resistance in this region has prompted evaluation of radical approaches to eliminate malaria which have relevance for other areas of the world with low malaria transmission. Targeted malaria elimination, even in the most remote and inaccessible areas, has been very effective.^35^, ^36^ The key to successful elimination is the support of village health workers in every village (usually 300‐800 people) to provide diagnosis of malaria with a rapid diagnostic test and then treatment with an effective ACT.^37^ In foci of higher transmission (sometimes called “hot spots”), where a significant proportion of the healthy population have asymptomatic parasitaemias, mass treatments with dihydroartemisinin‐piperaquine have proved very effective and well tolerated “accelerators” of elimination.^35^, ^36^ Mass screen and treat approaches are less effective and they are not recommended.^38^ However, whether these aggressive and more effective approaches to malaria elimination will be deployed by malaria control programmes remains to be seen.

### 1.5 Obstacle 1: The emergence and spread of antimalarial drug resistance

*P. falciparum* has developed resistance to all currently used antimalarial drugs, but there is substantial variation in the geographic distribution and in the degree of reduced susceptibility. The most resistant parasites are found in the Eastern GMS of Southeast Asia. Multidrug‐resistant *P. falciparum* is also prevalent in parts of South America. In Africa the most resistant *P. falciparum* parasites are found in Rwanda and Uganda. It is remarkable how drug resistance keeps emerging from these two relatively small areas of Asia and Africa. In general *P. falciparum* in Africa is more drug‐sensitive, with higher levels of resistance in East compared with West Africa.^39^ Resistance is generally less in the other malarias, although antifol resistance in *P. vivax* is widespread, and high levels of chloroquine resistance in *P. vivax* are found throughout Indonesia and Papua New Guinea.^40^ Antifol resistance in both *P. falciparum* and *P. vivax* results from stepwise accumulation of mutations in the *dhfr* gene which encodes the drug target dihydrofolate reductase (S108N, N51I, C59R). Sulphonamide resistance results from accumulation of mutations in the *dhps* gene encoding the drug target dihydropteroate synthase (A437G, K540E, A581G). In general, the more of these mutations there are, the more resistant is the *P. falciparum* infection.^41^ The highest level of antifol resistance is conferred by the *Pfdhfr* I164L mutation (found in Southeast Asia and South America). This mutation renders parasites completely resistant to pyrimethamine. Fortunately, except for Rwanda and adjacent Uganda, the 164 mutation has not established in Africa.^42^ Resistance to chloroquine and the structurally related antimalarials which interfere with haem detoxification results from mutations in the transporter *Pfcrt*, and to a lesser extent mutations in *Pfmdr* (notably N86Y, N1042D, S1034C and D1246Y). In the *Pfcrt* gene positions 72 to 76 are mutated in most *P. falciparum* (causing 4‐aminoquinoline resistance) with K76T being consistently mutant in the five major haplotypes (CVIET, SVMNT, SVIET, CVMNT and CVTNT).^40^ Recently mutations downstream from the chloroquine resistance locus have been strongly associated with resistance to the bisquinoline piperaquine.^23^, ^41^ Copy number increase in wild‐type *Pfmdr* is the main identified genetic association with mefloquine and lumefantrine resistance.^22^ Atovaquone resistance arises readily as a result of mutations in the mitochondrial multicopy cytochrome b gene (usually at position 268; Y268S or Y268N).

From a therapeutic perspective high‐level resistance in *P. falciparum* precludes use of chloroquine and sulphadoxine‐pyrimethamine alone or in combination in most areas.^1^ Amodiaquine alone is also not sufficiently efficacious in many parts of the tropics but still contributes significantly to efficacy in combinations. Artesunate‐amodiaquine remains efficacious in Central and West Africa. Significant resistance to mefloquine and piperaquine is prevalent only in the GMS of Southeast Asia. Fortunately, in these areas artemether‐lumefantrine and artesunate‐pyronaridine currently remain highly effective,^24^, ^43^ although both lumefantrine and pyronaridine are under significant selective pressure. As artemether‐lumefantrine is the most widely used antimalarial in the world, the emergence and spread of high‐grade lumefantrine resistance would have disastrous consequences for global malaria control.

### 1.6 Obstacle 2: Artemisinin resistance

**FIGURE 3:**
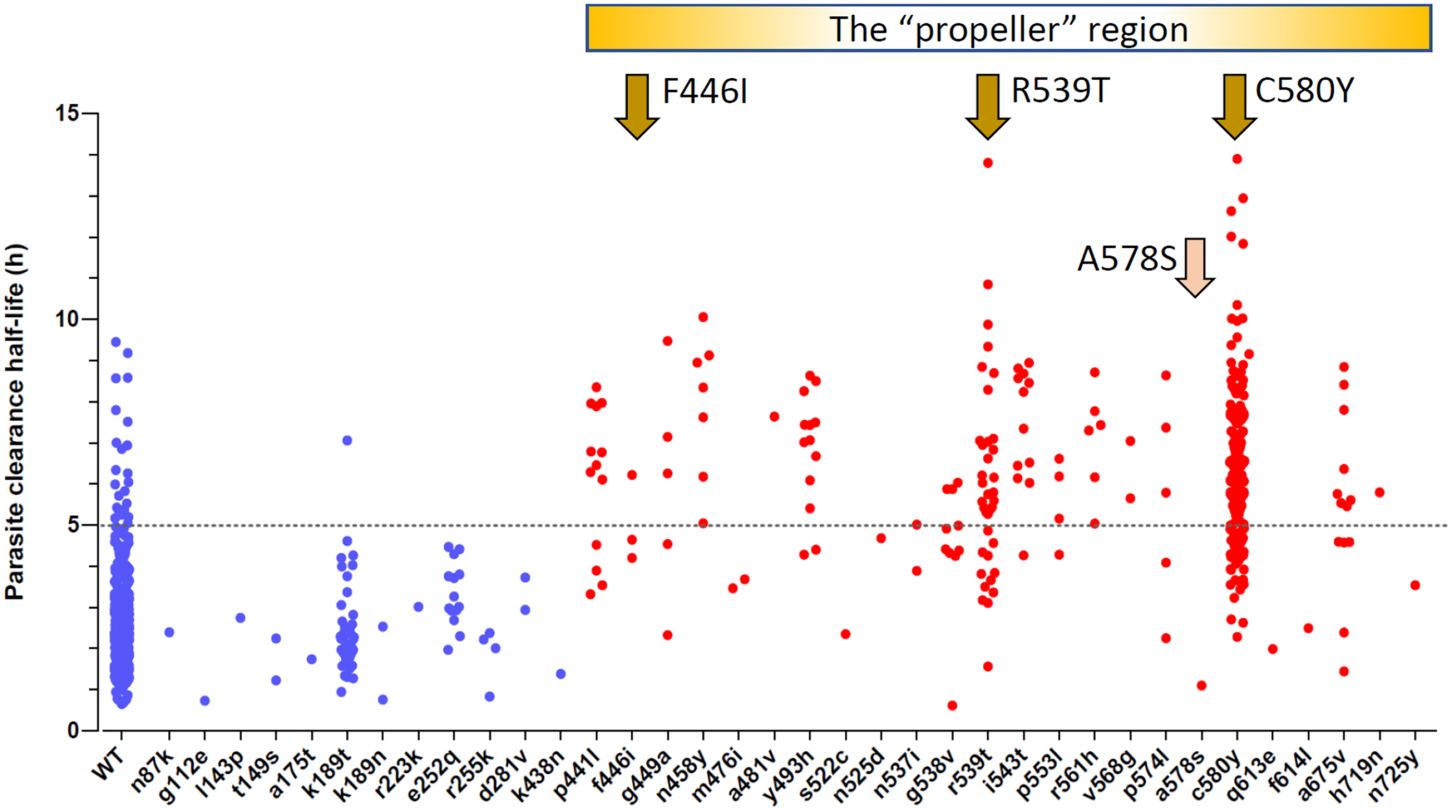
The parasite clearance half‐lives (PC_50_) associated with *Pf*kelch mutations in patients with acute falciparum malaria studied in the TRAC1 study.^20^ WT, wild type (note parasite clearance half‐life estimates can still exceed 5 hours in *Pf*kelch wild‐type infections). Mutations in the “propeller “region are usually associated with slow parasite clearance, the phenotypic hallmark of artemisinin resistance, although there is substantial interindividual variation and some mutations (A578S, pink arrow) are clearly not associated with artemisinin resistance. In the GMS parasite lineages associated with the F446I mutation have spread widely in Myanmar, and a lineage associated with C580Y was common along the Thailand‐Myanmar border before targeted elimination activities. In the Eastern GMS lineages associated with R539T and C580Y both spread, but in recent years a C580Y lineage (termed *Pf*Pailin) has dominated^44^, ^45^ (Figure 4). Modified from Ashley et al.^20^ with permission

**FIGURE 4:**
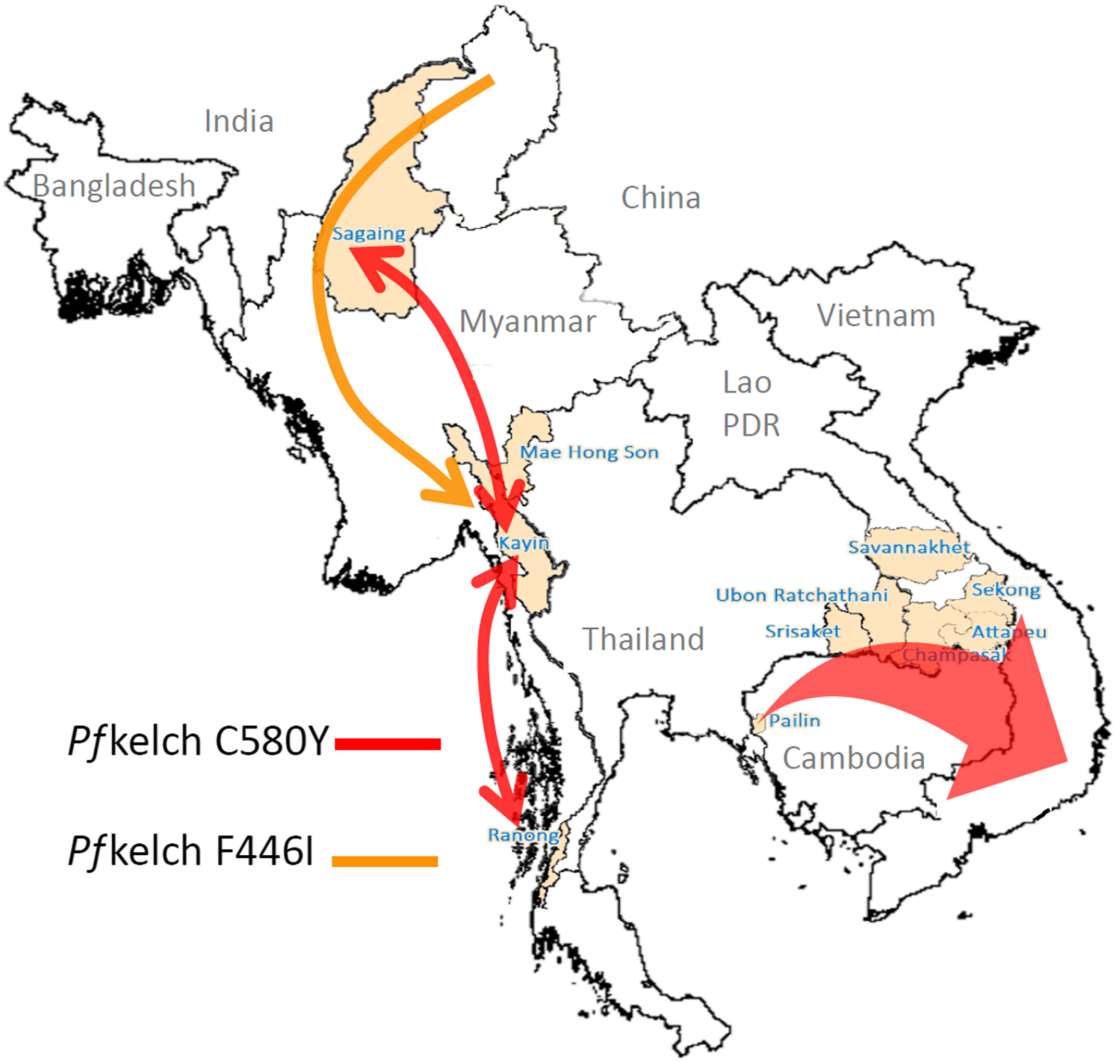
The spread of artemisinin‐resistant *P. falciparum* parasite lineages across the GMS. A single long *pfKelch* C580Y haplotype (from −50 to +31.5 kb either side of the *Pf*kelch gene), which emerged in Western Cambodia in or before 2008 (*Pf*Pailin), has spread across the Eastern GMS. In Myanmar C580Y parasites of a different lineage have spread widely but not dominated and a single *pfKelch* F446I haplotype, which probably originated in the north of Myanmar, has spread widely across the country. Modified from Imwong et al.^45^ with permission

It is an ominous precedent to the emergence and spread of artemisinin resistance in *P. falciparum* that the Eastern GMS is the same area from which resistance to chloroquine and sulphadoxine‐pyrimethamine arose and then spread to India and Africa (at a cost of millions of lives). Artemisinin resistance was found first near the Thailand‐Cambodia border. It manifests by slowing of parasite clearance which reflects reduced “ring‐stage” killing.^1^ In falciparum malaria the young ring‐stage parasites (in the first third of the 48 hour asexual life cycle) circulate in the bloodstream before the infected erythrocytes adhere to vascular endothelium (cytoadherence), a process called sequestration. This does not occur to a significant extent with the other human malarias. Sequestration is considered central to the potentially lethal pathology of falciparum malaria. The life‐saving benefit of the artemisinin derivatives (Figure 1) results from killing the ring‐stage parasites and thereby reducing sequestration.^12^ After starting treatment with an artemisinin derivative their pharmacodynamic effect is best measured in vivo from the log‐linear declines in parasite densities which follow a variable lag phase. The slope of this decline provides the parasite clearance rate and thus a parasite clearance half‐life (PC_50_). PC_50_ values over 5 hours are generally associated with artemisinin resistance^18^, ^20^ (Figure 3). When artemisinin resistance was recognized first multiple independent mutations were found in the *Pf*kelch gene propeller region, but in recent years successful artemisinin‐resistant parasite lineages have outcompeted the other parasites, and these dominant lineages have spread across large areas.^44^ In the Eastern GMS a parasite lineage bearing the C580Y mutation has predominated, whereas in Myanmar a lineage bearing the F446I mutation has spread over large distances^44^, ^45^ (Figure 4). The F446I mutation confers a lower degree of resistance (in terms of slowed parasite clearance) than many of the other propeller mutants. This may reflect a lesser fitness cost and thus greater competitive advantage in areas of higher transmission. These artemisinin‐resistant parasites have then acquired resistance to the ACT partner drugs piperaquine (in the Eastern GMS) and mefloquine (along the Thailand‐Myanmar border). The net result has been an increase in the rates of ACT failure,^21^, ^22^, ^23^ which has forced governments to change their first‐line malaria treatment policies. There is serious concern that these “fit” multidrug resistant parasites could spread westward, or that artemisinin resistance could emerge de novo elsewhere and derail global aspirations to control and eliminate malaria.

### 1.7 *Obstacle 3*: Underuse of primaquine

Primaquine is a very important antimalarial. It is recommended both as a single dose gametocytocide in falciparum malaria and in multiple‐dose “radical cure” regimens to prevent relapse in vivax and ovale malaria.^1^ But primaquine is underused. This is because of concerns over haemolytic toxicity in glucose‐6‐phosphate dehydrogenase (G6PD) deficiency.^46^ Gene frequencies for the X‐linked G6PD deficiency average 8‐10% in tropical areas (although because G6PD deficiency protects against *P. vivax* infections prevalences are lower in patients presenting with vivax malaria). Unfortunately, screening tests to identify G6PD deficient patients are not widely available. Relapses are recurrences of *P. vivax* or *P. ovale* malaria which follow complete cure of the blood stage infection. They derive from dormant parasite forms (called hypnozoites) which persist in the liver. Hypnozoites are resistant to all current antimalarial drugs except the 8‐aminoquinolines.^1^ Without radical cure relapse rates vary between 20% and 80%. They are often multiple, and they are a major cause of morbidity and mortality in higher transmission settings.^47^, ^48^ Primaquine has usually been given in 7‐ or 14‐day “radical cure” courses. As these regimens cause predictable haemolysis in G6PD deficient patients, G6PD testing is recommended before starting the radical cure regimen.^1^ The recent development of rapid G6PD deficiency screening tests is a significant advance which should enable wider safe use of primaquine for radical cure, and thereby make elimination a more achievable target.^49^ Recent very large studies confirm that the treatment durations, even for the higher dose primaquine regimens (total 7 mg/kg), can be condensed into a 1‐week course. With G6PD testing to exclude deficient patients, these are well tolerated. If these treatments are adhered to, radical curative efficacy is very high (>95%).^50^, ^51^, ^52^

In *P. falciparum* infections in low transmission settings single‐dose primaquine is used as a gametocytocide to reduce transmissibility of the treated infection. Until recently the dose recommended was 0.75 mg base/kg dose (45 mg adult dose). This was given in addition to the standard 3‐day ACT for treatment. Re‐evaluation of the transmission blocking dose‐response relationship for primaquine indicates that the same gametocytocidal effect is obtained with a dose three times lower (0.25 mg base/kg) with much less haemolytic risk. This obviates the need for G6PD testing so this has now become the recommended dose.^1^, ^53^

### 1.8 Advance 5: Tafenoquine

**FIGURE 5:**
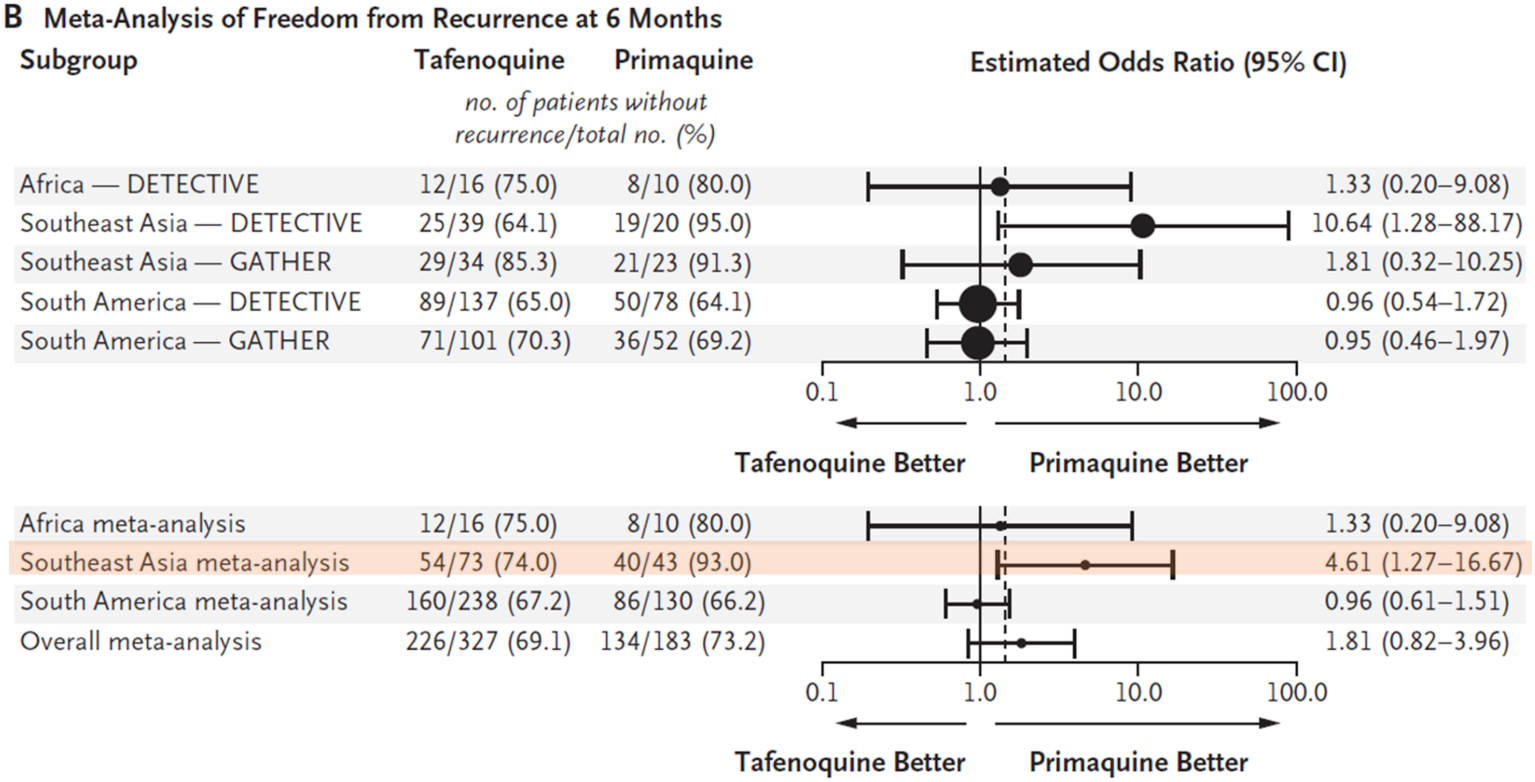
Radical cure of vivax malaria with tafenoquine. Individual patient meta‐analysis^55^ of freedom from recurrence of *P. vivax* malaria (relapse prevention) in the two tafenoquine pivotal phase 3 studies in adults. These compared tafenoquine single dose (300 mg) with a low‐dose primaquine regimen (15 mg base day for 14 days).^54^, ^55^ The dashed vertical line represents the prespecified noninferiority margin of an odds ratio for recurrence of 1.45 (tafenoquine *vs* primaquine). In Southeast Asia, which has high relapse rates, tafenoquine was significantly inferior (orange highlighting) to the low‐dose primaquine regimen (which is considered inferior to a high‐dose primaquine regimen of 30 mg base/day). Modified from Llanos‐Cuentas et al.^55^ with permission

For over 60 years primaquine has been the only widely available drug in the 8‐aminoquinoline class. In the past year, after a long and difficult gestation, the slowly eliminated 8‐aminoquinoline tafenoquine was finally registered and launched. Tafenoquine is a well‐tolerated single‐dose radical curative treatment which solves the problem of potentially poor primaquine adherence.^54^, ^55^ Like the other 8‐aminoquinolines, tafenoquine also causes oxidant haemolysis in G6PD deficiency. However, if a G6PD‐deficient patient haemolyses then the rapidly eliminated primaquine can be stopped, thereby limiting the consequent anaemia, whereas the slowly eliminated tafenoquine continues to cause haemolysis for weeks. Thus, tafenoquine has the advantage of simplicity and reliability of dosing, but at the expense of an increased risk of serious haemolysis. Currently available rapid screening tests identify individuals who have 30‐40% of normal erythrocyte G6PD activity.^49^ These tests identify all male hemizygotes and female homozygotes, but they do not identify the majority of female heterozygotes (whose blood contains a mixture of G6PD deficient and normal erythrocytes). These heterozygote females may haemolyse substantially on exposure to oxidant drugs. Safe use of tafenoquine therefore requires development and deployment of new simple quantitative G6PD screening tests which can identify accurately those individuals with <70% of normal red G6PD activity in blood samples (ie, all those at risk of significant haemolysis). These new tests are under development, but they are not yet ready for roll out. In East Asia and Oceania relapse is the main cause of vivax illness, a major contributor to morbidity and mortality, and a major obstacle to elimination. The dose of tafenoquine currently recommended (300 mg adult dose) is too low for *P. vivax* infections in this populous region, where a large proportion of the world's relapses occur. In the pre‐registration clinical trials relapse prevention with tafenoquine 300 mg proved inferior to a low dose of primaquine^55^ (Figure 5). Unfortunately, there are no plans currently to rectify this.

### 1.9 Obstacle 4: Medicine quality

Poor medicine quality is often ignored in discussions of disease control but the problem is massive, and it affects particularly the antimalarial drugs. In many countries the private sector is the main source of antimalarials and there is weak regulation of pharmaceuticals.^56^ A recent systematic review and meta‐analysis estimated that 12.4% of antibiotics and 19.1% of antimalarials in low‐income and middle‐income countries were substandard or falsified, with an estimated economic impact ranging from US$10 billion to $200 billion.^57^ Clearly this major obstacle to malaria control and elimination deserves more attention.

### 1.10 Obstacle 5: Political roadblocks and funding gaps

Discussion of roadblocks would be incomplete without considering the political dimension. Although malaria has a reasonable global profile in comparison with the “neglected tropical diseases” (at least until the COVID19 pandemic) it is often low in national health priorities, particularly in Asia and the Americas, where it is predominantly a disease of the poor or marginalized. Much of the funding for malaria control comes from international agencies such as the Global Fund to fight AIDS, TB and Malaria (GFATM) and the President's Malaria Initiative (PMI) or from bilateral donors. Whereas the world was doing very well in reducing malaria morbidity and mortality in the decade between 2005 and 2015, the total number of malaria cases has increased steadily since then.^58^ There has been no in‐depth analysis to explain this reversal, and no clear evidence that providing more funding without reforms will reverse this trend.

### 1.11 Advance 6: New antimalarials in development

Several new antimalarial drugs are in clinical development.^59^ These include the following:

- Cipargamin: a spiroindolone compound that is more rapidly acting (in terms of accelerating parasite clearance) than artemisinins. It inhibits PfATPase4.
- Artefenomel: a synthetic peroxide which is more stable and more slowly eliminated than the currently available synthetic peroxide arterolane.
- Ganaplacide: a potent imidazolopiperazine compound with an unknown mode of action.
- P218: a *dhfr* inhibitor with preserved activity against prevalent antifol resistant parasites.
- DSM265: a slowly acting dihydroorotate dehydrogenase inhibitor
- Ferroquine: an aminoquinoline compound with similarities to chloroquine but activity against chloroquine resistant parasites.
- MMV39004: a novel aminopyridine antimalarial compound that inhibits *Plasmodium* phosphatidylinositol‐4‐kinase (PI4K).

Most of these drugs are in phase 2 testing. If some of these compounds do proceed successfully through phase 3 studies and regulatory approval, likely in combinations, and these new combination therapies are well tolerated, effective and affordable, then they will be a welcome addition to the antimalarial armamentarium, but this is will not happen in the next few years.

## 2 CONCLUSION

There have been substantial advances in the control of malaria in the past 20 years. Insecticide‐treated bed nets and artemisinin combination treatments account for the majority of the millions of lives saved. Unfortunately, the promise of a highly effective and long‐lasting malaria vaccine, which dominated malaria research from the 1980s onwards, has not been fulfilled. Furthermore, with success in malaria control the sense of urgency has diminished and progress in many areas has stagnated. It is critically important that the efficacy of the current generation of antimalarial drugs is preserved for as long as possible, as the new compounds in clinical development are still several years from widescale deployment, and even then there is no guarantee that they will be as safe and effective as the drugs we have now.

### 2.1 Nomenclature of targets and ligands

Key protein targets and ligands in this article are hyperlinked to corresponding entries in http://www.guidetopharmacology.org, the common portal for data from the IUPHAR/BPS Guide to PHARMACOLOGY.

## COMPETING INTEREST

There are no competing interests to declare.
